# Progress in lactic acid bacterial phage research

**DOI:** 10.1186/1475-2859-13-S1-S1

**Published:** 2014-08-29

**Authors:** Jennifer Mahony, Francesca Bottacini, Douwe van Sinderen, Gerald F Fitzgerald

**Affiliations:** 1 School of Microbiology, University College Cork, Cork, Ireland; 2 Alimentary Pharmabiotic Centre, University College Cork, Cork, Ireland

**Keywords:** Bacteriophage, dairy fermentation, Antimicrobial, Genome sequencing

## Abstract

Research on lactic acid bacteria (LAB) has advanced significantly over the past number of decades and these developments have been driven by the parallel advances in technologies such as genomics, bioinformatics, protein expression systems and structural biology, combined with the ever increasing commercial relevance of this group of microorganisms. Some of the more significant and impressive outputs have been in the domain of bacteriophage-host interactions which provides a prime example of the cutting-edge model systems represented by LAB research. Here, we present a retrospective overview of the key advances in LAB phage research including phage-host interactions and co-evolution. We describe how in many instances this knowledge can be pivotal in creating real improvements in the application of LAB cultures in commercial practice.

## Background

Lactic acid bacteria (LAB) are a group of Gram-positive, non-sporulating bacteria encompassing several genera including among others, *Lactococcus, Streptococcus, Lactobacillus, Weissella, Leuconostoc, Enterococcus *and *Pediococcus*. They have been associated with food preservation for millennia, a property that is primarily mediated by lactic acid production as a result of hexose fermentation. Additional antimicrobial contributions can be made by a range of other metabolites produced at lower concentrations, depending on physiological and nutritional conditions, such as acetic, succinic and formic acids, acetaldehyde, ethanol and CO_2 _and bacteriocins.

When one considers the past 100 years of research on LAB, there is no doubt that the application of genomics from the early 1980s onwards has represented a major watershed. Prior to this, much of the research output was observational and descriptive with little mechanistic explanation of the phenomena in question (this of course was also the case with many other bacterial systems). There was an obvious scientific interest in elucidating key metabolic pathways, while the isolation and description of bacteriocins warranted the investigation into the molecular nature and mode of action of these antimicrobials in light of their potential medical and food preservation applications. In addition, the constant battle against bacteriophage infection stimulated efforts to obtain a better understanding of phage-host interactions.

With hindsight, the fact that plasmids play such a significant role in the functional activity of lactococci in particular proved to be very serendipitous. The peerless work of McKay, Klaenhammer and others in elucidating the role of plasmids in *Lactococcus lactis *was critical to the later development of gene transfer systems, the creation of vector plasmids and the general and progressive generation of the tools required for the genetic manipulation of these bacteria [1-4]. The explosion in research output across all members of the LAB, but particularly the lactococci and lactobacilli, was further stimulated by their essential role in a range of economically important activities ranging from dairy and other fermentation processes to their activities as probiotics and potential therapeutic delivery vehicles.

Over the past 30 years, the implementation of emerging 'omics' technologies such as genomics, transcriptomics, proteomics, metabolomics and when these are integrated, systems biology, to LAB research has resulted in the development of molecular tools that have been applied or formed the basis of development of such tools in other Gram-positive bacteria in the areas of protein expression systems, anti-microbial compound production and characterisation, glycobiology, cell envelope structure and (bacterio)phage-host interactions [5-14]. It is well documented that the advances in genomics and transcriptomics have realigned research focus away from gene mining towards the "interactomics" and rational exploitation of genomic data and this is no less the case in LAB research [15-18]. While each of these research foci has seen significant advances, perhaps the area exhibiting the most impressive developments is that of phage biology, to the extent that LAB phages and phage-host interactions have become an outstanding model organism for study in Gram positive bacteria. The scope of this review necessarily imposes a degree of selectivity on the topics that will be covered. Thus, based on the experiences of the authors and indeed the critical relevance of the topic from a commercial perspective, particular attention will be given to bacteriophages and phage-host interactions in lactococci.

### Phage-host interactions

Since their initial discovery in the 1930s [19], bacteriophages of LAB have been an obstinately persistent and costly problem in dairy fermentation processes. While aseptic procedures, culture rotation, sanitization and improved starter culture systems (such as the adoption of defined starters, the development and wide application of direct-to-cheese-vat cultures (or direct vat set (DVS)) have gone a long way to controlling phage infection, they still pose a serious risk especially in today's mega-scale production facilities where fermentations are performed on a very intensive and continuous basis.

As with many of the other technologically relevant activities of the LAB, research on phage and phage-host systems up to the 1980s was largely descriptive in nature, primarily due to the lack of incisive technologies that would provide an ability to unravel mechanisms underpinning these interactions. This is not to dismiss those studies which provided very significant information regarding lysogeny, phage ultrastructure, bacteriophage-insensitive mutants and phage-resistance systems. However, the adoption of molecular technologies from the 1980s and the subsequent application of genomics has proven to be spectacularly successful in clarifying the different phage taxa (particularly for phages of *L. lactis *and *Streptococcus thermophilus*), and has explained the impressive adaptability of phages, as well as providing an understanding of the nature of the infection process and elucidating the arsenal of phage resistance mechanisms that potentially susceptible hosts have evolved to combat infection.

The unearthing of the adaptive responses of phages and their hosts to host-encoded phage-resistance systems and to phage infection, respectively, has been a particularly intriguing area of study [20-22]. Similarly, the identification of novel genetic acquisition events which render phages increasingly fit in the dairy processing environment has been an especially rewarding outcome of this research. Knowledge of these adaptations may be applied in a predictive manner to understand the threat posed by phages as they evolve while also harnessing the hosts' response to the advantage of the dairy industry [23].

The most intensively employed LAB genera/species in the dairy industry as starter and adjunct cultures are *L. lactis, S. thermophilus *and *Lactobacillus *spp. [24]. Their industrial significance partnered with the availability of limited numbers of strains has accentuated the requirement for an in-depth understanding of the means by which LAB-phages infect their hosts to develop knowledge-based strategies to defend against infection. For this reason, phage-host interactions have been one of the major areas of phage biology to receive particular attention in the post-genomics era.

#### The role of genomics in LAB phage classification

**Figure 1:**
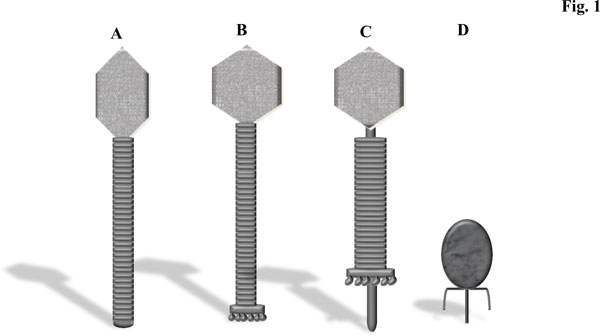
**Schematic representation of the known morphotypes of phages infecting LAB**. **1A **represents the prolate-headed *Siphoviridae *while **1B **represents the isometric-headed *Siphoviridae *phages. Members of the *Siphoviridae *family possess long non-contractile tails. **1C **displays the long contractile tail typical of *Myoviridae *phages. **1D **is a schematic highlighting the short non-contractile tails of the *Podoviridae *phages.

Over the past thirty years LAB-infecting phages have been classified by a number of means including electron microscopy, serotyping, DNA hybridisation, structural protein profiling and proteomic analysis and comparative genomic analysis [17,25-33]. The majority of phages infecting LAB belong to the family of *Siphoviridae*, which embodies a large group of phages with long, non-contractile tails and prolate or isometric capsids (Figure 1a &1b) [25,30]. The remainder belong to the *Myoviridae *(long, contractile tails) (Figure 1c) and *Podoviridae *(short, non-contractile tails) (Figure 1d) families, although these represent a small minority [34,35]. The dominance of the *Siphoviridae *phages may account for their high representation in model systems aimed at defining LAB phage-host interactions [36-38]. Lactococcal phages are currently grouped into ten taxonomic groups based on morphology and DNA homology, and of these the P335, 936 and c2 species (all *Siphoviridae *phages) are the most frequently encountered in the dairy industry [25]. All currently known phages of *S. thermophilus *belong to the *Siphoviridae *family and were until recently classified into two groups based on their mode of packaging (cohesive ends termed *cos *phages or headful packaging termed *pac*) [30]. Interestingly, a third group represented by a phage with a novel genetic lineage (5093-like phages) has recently been described [39]. In contrast, classification of *Lactobacillus *phages is much more complex due to the genetic diversity that they display and it has been suggested that they should be typed by host range and morphology, and by genetic relatedness at the intra-species level [40].

While the traditional methods of classification of LAB phages relied on a range of techniques, many of which were labour and time-intensive and occasionally imprecise, it is now possible to sequence phage genomes using an array of evolving and improving technologies with relatively short turnaround times. This has led to a dramatic increase in the amount of phage genomic data available with which one can rapidly compare and (genetically) classify phages. This has formed the basis for the development of many multiplex PCR tools for the rapid identification and speciation of LAB phages, particularly for those known to be regularly infecting *L. lactis *[41,42]. The use of proteome-based taxonomic systems such the "Phage proteomic tree"[33] demonstrates that "omics" data can be applied to useful taxonomic schemes that may harmonise previously scattered approaches to this important issue.

While the dominance of particular lactococcal phage species has long been known, it was not until the end of the last decade and the beginning of this decade that we now fully appreciate the genetic diversity of these individual species. For example, there are 45 fully sequenced members of the 936 phage species and while there are localised regions of variability, their genome sequences and their overall genomic architecture are highly conserved [43,44]. In contrast, there are 10 fully sequenced members of the P335 phage species (excluding prophages) and these are now categorized into four subgroups based on sequence homology and baseplate type [45]. While the P335 genome architecture and modular organisation is well-conserved, the functional modules may vary considerably with respect to their sequences and represent a "melting pot" of genetic information. Given that many of these phages are temperate, it is perhaps unsurprising that they are observed to be more diverse as they may acquire genetic elements from their hosts, and indeed this diversity and complexity would provide justification for further sequencing programmes of P335 phages. The c2 phages are represented by only two fully sequenced members, c2 and bIL67 [46,47], and both appear to be very similar genetically. Although this species dominated in early phage isolation studies, it appears that they have become less problematic recently and for this reason we will focus on the dominant 936 and P335 species in this review.

**Figure 2:**
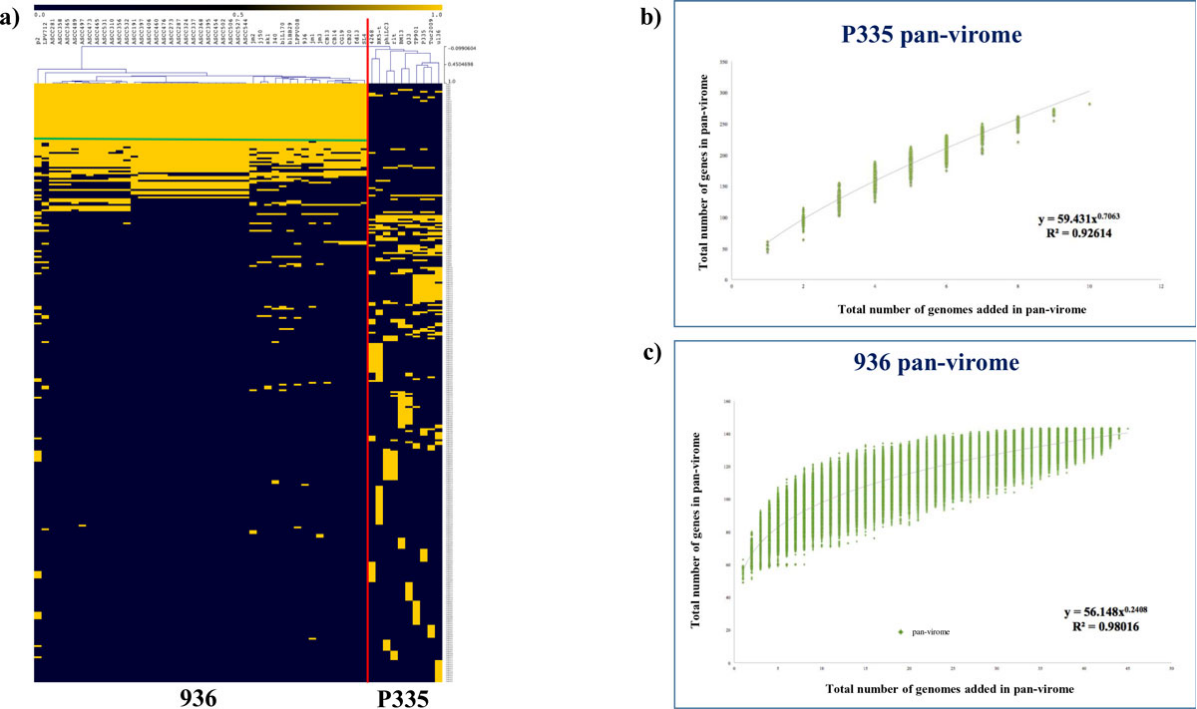
**Pan-virome analysis of the P335 and 936 phage species**. **2A) **Heatmap showing the hierarchical clustering analysis conducted on the P335 and 936 data sets. The separation achieved between the two species is indicated by a vertical red line, while the 936 common genes (core genome) area is indicated by a horizontal green line. **B & C) **Accumulated number of P335 (b) or 936 (c) pan-virome genes, respectively, plotted against the number of P335 (b) or 936-species (c) phage genomes added. The deduced mathematical function is also indicated.

Understanding the complexity or conservation of a given phage species is vital to define the necessity of future phage genome sequencing projects and these aspects can be well resolved by the application of computational methodologies involving comparative genomics and pan-genome analysis on the available P335 and 936 sequences mentioned above. The relatively recent concept of pan-genome (or pan-virome if it applies to viral genomes) analysis has enjoyed considerable application as a means to describe genomics of bacterial species, facilitated by the increasing number of Next Generation Sequencing (NGS) projects that have been undertaken. Pan-genome analysis considers a species as a single entity composed of the entire set of genes present in each representative (also called pan-genome), which can be further divided into two classes of genes, i.e. those commonly present in all (core-genome) and the unique genes or those shared between a few members (dispensable genome) [48]. The P335 pan-virome, which is based on the sequenced representatives of this phage species, displays a considerable level of genetic diversity (Figure 2a). The open state (indicated by the "exponential" appearance of the graph that has not yet reached a plateau) of the pan-virome function indicates that this diversity has not yet been fully captured by the currently sequenced P335 genomes (Figure 2b), as it predicts that with the addition of new P335 genomes a considerable number of new genes will be added to the P335 pan-virome. In contrast, given the high degree of conservation of phages belonging to the 936 species (Figure 2a) and the almost "closed state" (indicated by the plateaued appearance of the graph reflecting that newly sequenced genomes of this phage species do not contain genes that had not been found in previously sequenced phages of this species) of the pan-virome function achieving a plateau status (Figure 2c), sequencing of additional 936 phage genomes is not likely to uncover new genes, although phages with new combinations of previously found genes may still be out there.

#### Host-encoded receptors of LAB-infecting phages

The primary interaction between phages and their hosts is based on the recognition of a host-encoded receptor by a structure at the distal end of the phage tail known as the receptor binding protein (RBP). The molecular players involved in this initial physical connection between LAB phages and their hosts have been the subject of intense scrutiny, particularly over the past decade. While there are multiple levels at which phages may interact with their hosts involving various different host and phage structures, these may be simplified into groups based on their receptor material: protein or carbohydrate (or (lipo)teichoic acid).

**Figure 3:**
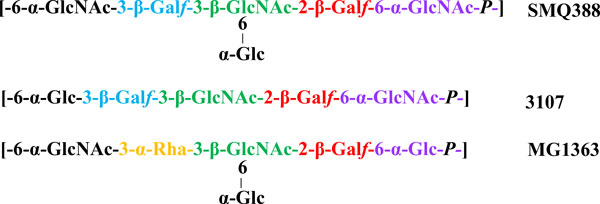
**Figure adapted from Chapot-Chartier et al**., 2010 [51] and Farenc et al., 2014 [53] highlighting the structure of the cell wall polysaccharide of *L. lactis *SMQ388, 3107 and MG1363. The relatively conserved rightmost trisaccharide coloured in green, red and purple represents the proposed receptor for the lactococcal phages infecting these host strains i.e. 1358, TP901-1 and p2, respectively.

The lactococcal 936 and P335 phages are believed to recognise cell surface-located saccharidic moieties [49,50], which are part of a so-called pellicle or cell wall polysaccharide (CWPS) [51]. The CWPS of three lactococcal strains (MG1363, 3107 and SMQ388) have been defined as a phospho-polysaccharide [51-53] composed of repeating subunits of a phospho-penta/hexasaccharide linked by phosphodiester bonds (Figure 3) [51]. Intriguingly, within these repeating structures is a conserved trisaccharide component that is believed to be a common receptor for phages p2, TP901-1 and 1358 of the 936, P335 and 1358 species, respectively [53]. The operon encoding the biosynthetic machinery for this CWPS has been identified as a 20 - 30 kb genomic region and mutations in genes within this operon render the host strain insensitive to infection by 936-type phages [54]. Genetic diversity within this cluster of genes has been associated with potential diversity in the biochemical structure of the CWPS of lactococcal strains and comparative genomic analysis of this cluster within sequenced strains has led to the identification of three CWPS-specifying (geno)types (type A, B and C) based on genetic elements that are specific to that group [10]. Furthermore, a link has been established between the CWPS type of the lactococcal host and the phylogenetic grouping of 936 phages RBPs [10]. Thus, it is possible to predict the sensitivity of particular lactococcal hosts to particular subgroups of 936 phages. This is the first such molecular tool for defining relationships between collections of phages and strains. Further exploration of the biochemical characteristics and compositional structure of the CWPS of lactococcal strains will undoubtedly provide key information regarding the saccharidic components that act as receptors for these phages. It is likely that many other species of lactococcal phages and indeed those of other LAB genera that are still underrepresented in current phage/host studies, employ similar saccharidic receptors. As the number of phage/host genome sequences becoming available increases, it may be possible, through comparative genomics and mutational analyses, to identify the operons/genes involved and thus to develop predictive tools, such as the PCR-based method described for lactococcal phages and hosts, for a wider range of LAB hosts and phages in order to provide a risk assessment of phage infection.

In the dairy industry, the development of bacteriophage-insensitive mutants (BIMs) [24,55] of important fermentation strains is a crucial short-term measure used to counter the phage problem. For decades this has been a practice that has been performed without any significant understanding of the underlying reasons for the associated phage-resistance. Perhaps now it is possible to suggest that mutations in/diversification of the CWPS-specifying gene cluster may be responsible for at least some of the elements underpinning the acquired phage-resistance in lactococci. While knowledge of the potential reason for the phage-resistance in BIMs is unlikely to herald the end of the 'BIMs' approach to avert phage-mediated problems it will most likely facilitate a knowledge-based strategy to the design of the next generation of robust, technologically appropriate and stable BIMs.

In contrast to saccharide-recognizing phages, the lactcococcal c2 phages recognise a protein component on the surface of their host cell [56]. The protein involved is termed the "**P**hage **I**nfection **P**rotein" (PIP) and is a large membrane-associated protein analogous to the YueB receptor for the *Bacillus subtilis *phage, SPP1 [57]. This gene encoding this protein appears to be universally present in lactococcal genomes, thus accounting for the typically broad-host range of these phages. As expected, removal of the genetic material encoding the predicted membrane-spanning domains of PIP prevents phage infection of the resulting PIP^- ^lactococcal strain, a fact that may be harnessed to prevent proliferation in the dairy fermentation setting, assuming that the growth and technological characteristics of the PIP^- ^strain are not negatively affected [58]. This could be achieved by genetic modification (GM) as previously described [58] or by means of a non-GM method through the isolation of BIMs of lactococcal strains resistant to c2 phages after exposure of the parent culture to the phage. The resulting BIMs (or many of) presumably possess a deletion/alteration of the PIP-encoding gene. Once it is established that such a deletion does not affect the BIM's ability to be used as a starter culture, such strains can be applied as "natural derivatives" of the parent strain.

The *Lactobacillus delbrueckii *ssp. *lactis *strain ATCC15808 and its infecting phage LL-H is the most thoroughly investigated phage-host model system for the *Lactobacillus *genus. Interestingly, LL-H is one of very few reported phages infecting a Gram-positive bacterium employing lipoteichoic acids (LTAs) as a receptor [36,59,60]. In this phage-host interaction system, incubation of purified LTAs with phages LL-H, Ads-5 (a host range mutant derived from LL-H) or JCL1032 caused more than 90% reduction of phage infection [60]. While LL-H has served as an excellent model system for the interactions of *Lb. delbrueckii *phages and hosts, a significant knowledge gap still remains in understanding the interactions between *Lactobacillus *strains and infecting phages given the genetic diversity found within members of this bacterial genus and their viral parasites. While this poses a significant scientific challenge, it also presents an exciting opportunity and novel area of phage-host interactions that deserves research attention.

### The role of divalent cations in the phage infection process

**Figure 4:**
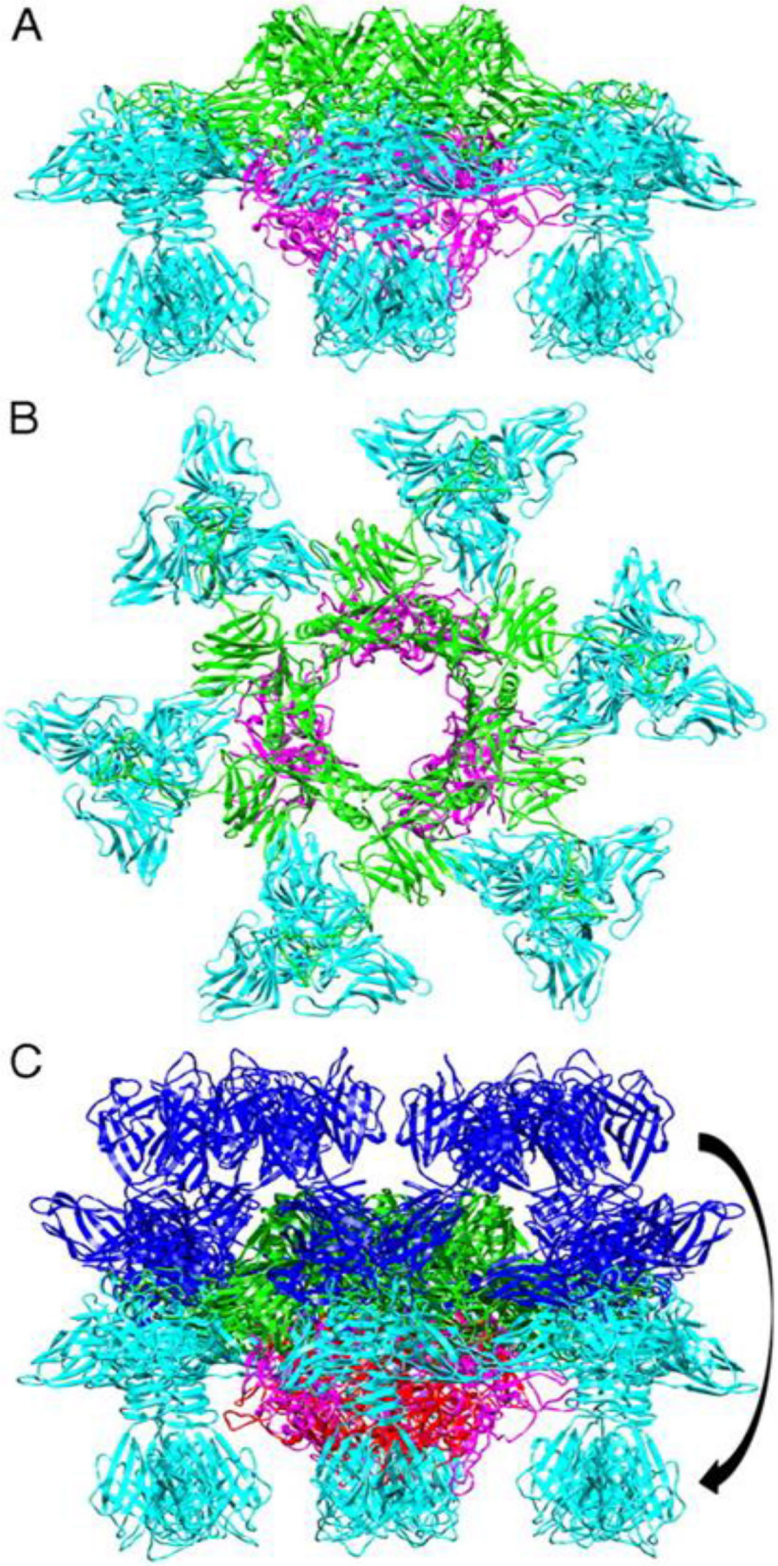
**The crystal structure of the "heads-down" conformations of *L. lactis *phage p2 baseplate**. **(A) **Side view in ribbon representation of the "heads-down" conformations of *L. lactis *phage p2 baseplate. **(B) **View from top (ORF15, green; ORF16, pink; ORF18 (RBP), blue). **(C) **Superposition of the rings formed by the N-terminal domains of ORF15. ORF18 (RBP) trimers have undergone a 200° rotation downwards [63]. This figure has been taken from Sciara et al., 2010 with permission [63].

The dairy industry and its associated starter culture technologists have long held the view that calcium is required for phage infection, at least by some phages. It is for this reason that phage-inhibitory medium (PIM) was developed incorporating phosphate in a whey-based bulk starter medium to "mop up" excess divalent cations during the propagation of the starter culture prior to inoculation of the milk [61] (For a review, see [62]). While this was a rational approach, it is not always successful, a phenomenon that could not be fully explained until quite recently. Thus, the discovery by the Cambillau group [63] describing the activation of the baseplate of a 936 species phage, p2, in the presence of calcium or other divalent cations was particularly apposite. In this study, the crystal structure of the baseplate of p2 was analysed and it was observed that in the absence of calcium the receptor binding region of the baseplate was flipped upwards facing the capsid rather than the host cell, an orientation that seems counterintuitive. However, upon calcium addition, the baseplate realigns itself through a 200° downward movement to face the host. This intriguing baseplate activation may represent one possible explanation for the success of the 936 phages as the dominant species in the dairy industry as it will only be activated in a calcium-rich environment, such as milk, while remaining in a "closed" yet stable state until that situation occurs (Figure 4).

**Figure 5:**
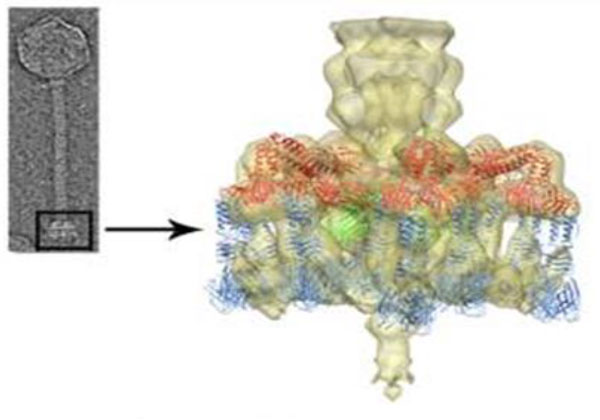
**Structure of the *L. lactis *phage TP901-1 baseplate (i.e., host adsorption machinery)**. This figure is adapted from Veesler et al., 2012 with permission [64]. **(Left) **Negatively-stained electron micrograph of a TP901-1 virion. **(Right) **Close-up view of the phage baseplate X-ray structure fitted in the adsorption device three-dimensional electron microscopy reconstruction (the region is highlighted by a black square on the micrograph). The baseplate is formed by 18 copies of BppU (red) arranged around a central Dit hexamer (green) and holding eighteen trimeric RBPs (receptor-binding proteins, blue). The baseplate is permanently observed in the RBP downward-facing or "infection-ready" orientation.

For the P335 phages, there is a mixed requirement for divalent cations. For example, TP901-1 and ϕLC3 do not require calcium for phage infection while Tuc2009 infection positively correlates to the concentration of calcium present in the medium [64]. The finding that TP901-1 does not require calcium for infection is in agreement with its permanent "infection-ready" conformation with the baseplate facing downwards in the presence or absence of calcium (Figure 5).

These observations represent the first crucial evidence explaining why phage-inhibitory media do not successfully prevent all phage infections, and in fact provide an exquisite example of the role of structural biology in addressing a long-standing conundrum relating to the variable performance of PIM when used in commercial practice.

To further ascertain the requirement for divalent cations in the infection process representative members of six additional species of lactococcal phages, as well as some previously characterised phages of the P335 and 936 species have been assessed (or to verify) for their requirement for calcium. Plaque assays incorporating a range of calcium chloride concentrations from 0 - 10 mM and in the presence of the chelating agent EDTA were performed. In this study, only phage 1706 [65] was found to require calcium for plaque formation, although it should be noted that plaque visibility was significantly improved for phage 949 [66] and members of the 936 phage species in the presence of calcium (unpublished data). Phage 1706 was unable to produce plaques with medium containing less than 10 mM calcium chloride. The c2 phages as well as Q54 [67] and P087 [68] infect their host with comparable efficiency in the presence or absence of calcium, or in media containing EDTA (unpublished data).

### Identification of phage-encoded receptor binding proteins of LAB-infecting phages

Phage genome sequencing and subsequent bioinformatic analysis has led to the presumptive identification of RBP-encoding genes [38]. The isolation of chimeric phages containing a "swapped" receptor binding domain allowed the identification of the gene responsible for host recognition in *S. thermophilus *phages DT1 and MD4 [38]. Employing a similar strategy, the RBPs of the lactococcal P335 species phages, TP901-1 and Tuc2009 have also been identified [37] as were those of the lactococcal 936 phages, sk1 and bIL170 with confirmation by immunogold labelling electron microscopy [49]. Furthermore, immunogold staining has also been central to the identification of the genetic determinants of c2 phages responsible for the interaction with their protein receptor, PIP, i.e. *l10 *and *orf31 *of phages c2 and bIL67, respectively, [46,47].

Most recently, chimeric phage development has been applied to identify the RBP of *Leuconostoc pseudomesenteroides *phages, P793 and LN04 [69]. Bioinformatic analysis and domain exchange have not only permitted the identification of the RBP, but also the specific protein domain that is required for this interaction to occur. It is now well established that the amino-terminal portion of these proteins may be well-conserved within a group or species of phages, while the carboxy-terminus may vary considerably, and it is these unique or variable regions that dictate the specific interactions with the host [37,38,49]. Given the hypervariability associated with these host-specificity domains, it is perhaps unsurprising that the host range of these phages is generally quite limited and specific.

Significant progress has been made in the structural analysis of the base plate and associated RBPs of lactococcal 936 and P335 phages which has considerably enhanced our understanding of the mechanisms of interaction with the receptor and in specific cases it has led to the identification of the saccharidic target compound on the host cell surface [70]. The 936 phage p2 was the first lactococcal phage RBP to be analysed in this manner and this work revealed three domains of the RBP structure, namely the head (receptor binding domain), neck and shoulder domains [71]. This was followed by studies of chimeric RBPs of the 936 phages bIL170 and p2, which showed that while there is little sequence homology between the head domains of these two phages, a structural similarity is retained [72,73]. This supports genomic studies which suggest that while sequence similarity may not be maintained, gene order and the architecture of functional modules is conserved [27,74]. The observation of structural conservation in the absence of sequence conservation has been observed not only between members of the 936 species but also between the 936 and the P335 phage TP901-1 [73]. For example, it is now possible to predict which genes of P335 or 936 phages may encode the initiator complex or baseplate components such as the distal tail protein (Dit), the tail-associated lysin (Tal) and baseplate/RBP components once the RBP itself has been defined, even in the absence of sequence similarity. Such elements may then be exploited for structural or functional analysis [12,70,75-78]. Indeed, the finding of structural conservation goes far beyond lactococcal phage species as similarities have been observed between the head domains of phage p2 and those of mammalian viruses including adenoviruses and reoviruses [71]. Using the information derived from such structural studies, proposals for assembly pathways of the phage tail and its baseplate components of P335 and 936 phages have been established based on Western blot analysis [79] and mass spectrometry [80], respectively. It is now proposed that Dit acts as a central hub around which the baseplate is formed and to which the Tal is attached to act as the initial puncturing device (For extensive reviews on this topic, see [11,78,81]).

Structural biology studies such as these represent one of the major areas of success in phage research in recent years and have transformed our previously primitive view of phage-host interactions and filled many of the knowledge gaps that were difficult to resolve by other methods including the identification of the saccharide binding sites.

### Phage-host Co-evolution

The 1980s and 1990s represented the golden era of the identification and characterisation of an impressive array of phage-resistance systems from abortive infection and restriction-modification (R/M) systems, through to DNA injection blocking and adsorption blocking [82-84]. The concept of phage-host co-evolution is not a new one; however, distinct advances have been made in this area in the case of LAB phages and hosts, particularly in relation to the CRISPR (Clustered Regularly Interspaced Short Palindromic Repeats)-driven evolution of strains and phages of *S. thermophilus *[20]. CRISPR systems are widespread among strains of *S. thermophilus *and *Lactobacillus *spp. [85,86], while only one report of a plasmid-encoded CRISPR exists for lactococci [87]. These phage-resistance systems provide acquired immunity against phages and incoming foreign DNA that can also include plasmids [86]. Spacers of short DNA segments are acquired from the infecting phage and provide immunity against subsequent exposures to the same phage. Noteworthy however, is the relative ease with which these systems may be overcome by phages as only a single nucleotide substitution is required to bypass the immunity system [20].

The justified attention that has been paid to CRISPR-mediated phage-resistance has unearthed many interesting findings. Among these is the fact that CRISPR systems may account for the relative ease with which BIMs of *S. thermophilus *are generated in comparison to their non-CRISPR containing lactococcal counterparts. CRISPR-mediated BIMs may occur at relatively high frequencies compared to non-CRISPR-mediated BIMs, which in the latter case may require host genome mutation or IS element repositioning. For decades, it has been known that it is more difficult to generate spontaneous BIMs of *L. lactis *compared to *S. thermophilus*; however, with the current knowledge of CRISPR systems in lactic streptococci, it is now possible to explain this phenomenon.

In addition to CRISPR defence systems, co-evolution studies of phages that have overcome abortive infection (Abi) systems have also been described, most abundantly for lactococcal phages, and through the isolation of these so-called escape mutant phages, it has been possible to identify the genes targeted by various Abi systems, including AbiQ, AbiT, AbiV [21,22,88,89]. The molecular targets of these abortive resistance systems are dispersed throughout the relevant phage genomes depending on the mechanism of the individual Abi in question. Sequence analysis of such escape mutant phages provides information which is vital to develop a basic understanding of the *modus operandi *of Abi systems. In recent years, the finding of novel Abi systems has slowed dramatically in comparison to the 1980's and 1990's; however, research attention has since shifted towards mechanistic studies, which will be invaluable in developing the next generation of knowledge-based defences against phages in the dairy industry and beyond.

While *S. thermophilus *strains appear to rely quite heavily on CRISPR systems to combat phage attack, lactococci on the other hand engage in "stacking" of phage-resistance systems encoded both on their plasmids and on their chromosomes [90]. Through genomic analysis, it has become clear that lactococci perhaps compensate for the lack of CRISPR-based immunity by carrying a suite of armour that will target different phage species/isolates, and may further strengthen their defences by trying to interrupt the phage infection process at multiple stages. For example, lactococcal plasmids have long been known to stack resistance/modification (R/M) systems and this may also be paralleled on the chromosome [90]. The first type II R/M system to be defined, ScrfI, was found on the genome of the lactococcal strain UC503 [83,91] and this initiated the search for other such systems. Akin to Abi systems, the identification of novel lactococcal R/M systems has lost momentum in recent years although their more recent identification in other genera, e.g. bifidobacteria [92], is notable. In addition to host-encoded phage-resistance systems, prophage-encoded systems have been characterised over the past decade predominantly including DNA injection blocking systems epitomised by the lactococcal superinfection exclusion system Sie_2009 _encoded by the temperate lactococcal phage Tuc2009 [93]. Since the identification of this system, several others were described in both *L. lactis *and *S. thermophilus *[94,95]. The structures of phage-resistance proteins, including those of the *S. thermophilus *superinfection exclusion system, Ltp_TP-J34 _and the Abi system AbiQ, have been resolved [89,96]. These data shed new light on matters which were hitherto not understood and provide a unique angle from which we can begin to understand the subtleties of the relationship between phage and host.

## Conclusions

LAB research has advanced spectacularly over the past 30 years and there are many areas in which these bacteria provide model systems for other genera, particularly Gram-positive bacteria. This is particularly true in the case of LAB phage research. While technological advances in the areas of genomics, transcriptomics and recombinant protein production have all contributed to the progress of LAB phage research, it is obvious that several knowledge gaps still remain. For example, research has focused quite heavily on the industrially significant genera (*L. lactis *and *S. thermophilus*), while the interactions of other genera remain poorly defined, yielding scope for future studies. While it is also the case that transcriptomic analysis of lactococcal hosts has been performed to assess the response of the cell to phage infection by member of the c2 and P335 species [15,97], the specific process and detail of LAB phage genome replication and host responses to infection and phage replication remain relatively unclear. Therefore, future transcriptomic studies perhaps should focus on standardising the approaches used (e.g. multiplicity of infection, exponentially growing cells or stationary phase, etc.). Additionally, post-translational modification of host proteins should also be considered as another level of host-response in cases where global shut-down of the host is observed post-infection as suggested by Lavigne and colleagues [98].

Perhaps, it is evident that while significant advances have been made in defining the initial interactions between LAB phages and their hosts, much remains to be discovered in aspects relating to the mechanics of DNA injection and replication, which may represent the next generation of LAB phage research in the post-genomics era.

## Competing interests

The authors declare that they have no competing interests.
